# Scalable interconnection using a superconducting flux qubit

**DOI:** 10.1038/s41598-024-65086-1

**Published:** 2024-07-16

**Authors:** Daisuke Saida, Kazumasa Makise, Mutsuo Hidaka

**Affiliations:** 1 Fujitsu Limited, 1-1, Kamikodanaka 4-chome, Nakahara-ku, Kawasaki, Kanagawa 211-8588 Japan; 2 National Institute of Advanced Industrial Science and Technology, Ibaraki, Japan; 3 National Astronomical Observatory of Japan, Tokyo, Japan

**Keywords:** Electrical and electronic engineering, Qubits

## Abstract

Superconducting quantum computers are rapidly reaching scales where bottlenecks to scaling arise from the practical aspects of the fabrication process. To improve quantum computer performance, implementation technology that guarantees the scalability of the number of qubits is essential. Increasing the degrees of freedom in routing by 2.5-dimensional implementation is important for realizing circuit scalability. We report an implementation technology to overcome the scaling bottlenecks using a reliable connection qubit with a demonstration of quantum annealing. The method comprises interconnection based on quantum annealing using a superconducting flux qubit, precise coupling status control, and flip-chip bonding. We perform experiments and simulations with a proof-of-concept demonstration of qubit coupling via interconnection using a flux qubit. The coupling status is strictly controllable by quantum annealing. A low-temperature flip-chip bonding technology is introduced for the 2.5-dimensional interconnection. The superconducting flux qubit, formed across two different chips via bumps, is demonstrated for the first time to show a state transition like that in a conventional qubit. The quantum annealing flux qubit and flip-chip bonding enable new interconnections between qubits. A perspective on the possibility of applying this technology to the connection between gate-type qubits is described.

## Introduction

**Figure 1:**
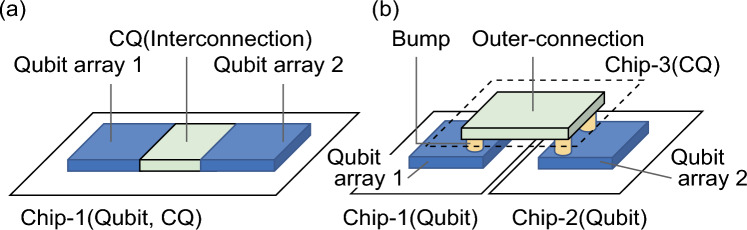
**(a)** Proposed scheme for the two-dimensional integrated quantum processor. Each unit lattice, composed of several flux qubits, is connected by the connection qubit (CQ). Unit lattices are fabricated in the same chip. (**b)** Proposed scheme for 2.5-dimensional integrated quantum processor. Qubits constructed in different chips (Chip-1, Chip-2) are connected to the CQ (Chip-3) by flip-chip bonding.

Over the past decade, superconducting quantum circuits have become an important foundation for quantum computing scalability. Transmon qubits^1,2^, one of the most promising candidates for scalable quantum circuits, continue to increase the number of qubits implemented in a quantum computer^3–5^. The generic gate-type qubits have two-dimensional variants^6,7^ and three-dimensional structures^8–11^. However, solving practical problems requires millions of error-correctable qubits or more, and thus there is still a tremendous challenge in fabricating and integrating qubits. An alternative method that is particularly suitable for solving combinatorial optimization problems is using quantum circuits for quantum annealing (QA). The advantages of quantum computation using QA over conventional methods are still under investigation, but practical demonstrations will be available in the near future^12–21^. A superconducting flux qubit is used in QA^12–14^. Nonlinear inductance (*L*) of a Josephson junction (JJ) is modulated by applying an external flux to the superconducting flux qubit. In QA, the shape of the qubit energy potential is time-modulated to obtain a solution corresponding to the ground state of the problem Hamiltonian. The ground-state spin logic^22^ allows us to obtain the Hamiltonian for the invertible logic^23,24^ when the input and output relationships are expressed by Boolean logic gates. If the optimization problem can be expressed using logic circuits, the circuits can be converted to the problem Hamiltonian. We have designed superconducting quantum circuits according to the Hamiltonian and experimentally demonstrated their functionality^21^. This method is expected to increase the solution accuracy because the implementation of the problem Hamiltonian as the converted logic circuits does not require a spectrum change in the excited state. Moreover, fewer qubits are required for implementing the Hamiltonian compared with the number on a prefabricated unit lattice, such as a chimera graph^12^. The quantum circuit size can be expanded by interconnecting the lattices (Fig. 1a). In this work, we consider qubit interconnection that can connect long distances of millimeters with a strong coupling force of tens of picohenries or more. This will allow us to expand the number of qubits within the same chip. In extending the scale of quantum computers, we must focus on the fact that the fabrication process is reaching a scale where the variations of qubits within the chip are becoming uncontrollable. A countermeasure is to fabricate qubit circuits on separate chips, where qubits retaining high characteristics are fabricated at high yields and assembled three dimensionally (Fig. 1b). The two chips can then be bonded together via bumps using flip-chip bonding.

We consider a long-distance outer-interconnection using a superconducting flux qubit [namely, a connection qubit (CQ)] with precise coupling controllability. The method comprises the following three techniques: interconnection based on QA using the CQ, precise coupling status control, and flip-chip bonding. To accomplish this idea, an experimental demonstration is needed to show the long-distance interconnection using the superconducting flux qubit. We demonstrate that the superconducting flux qubit can connect long distances of millimeters with a strong coupling force of tens of picohenries or more using QA. Additionally, clarifying how to control the interconnection status precisely is necessary. Moreover, Fig. 1b shows that the top and base chips are bonded together via bumps using flip-chip bonding. Low-temperature resistance measurements with connected superconducting wires consisting of tens of thousands of bumps have been reported^25^, anticipating fabrication of large quantum circuits with a 2.5-dimensional implementation. However, in an actual circuit, there are multiple bump diameters in the fine pattern and the surrounding sections, resulting in uneven bump heights due to surface tension differences. Furthermore, heating during the bonding process must not change the qubit characteristics. The application of flip-chip bonding, where the qubit and the readout circuits are formed on separate chips and connected by bumps, has been reported^25–29^. However, the qubit itself has not been fabricated by flip-chip bonding, and this method would minimize the occupation area in the routing between unit cells. By forming the interconnection qubit with flip-chip bonding, we can provide a new way for qubits to interact, while adding coupling status controllability.

In this work, the three key technologies of interconnection based on QA using the CQ, tunability in the coupling, and flip-chip bonding, are demonstrated in turn. First, the qubit interconnection through QA using the CQ is experimentally demonstrated. Second, we clarify how to control the coupling between qubits. Third, we introduce the flip-chip bonding method for the 2.5-dimensional implementation that does not affect the qubit characteristics. The superconducting flux qubit constructed across different chips via bumps shows the same operation as a conventional qubit. As a possibility for solving the bottleneck of scaling in gate-type qubits, a perspective on applying combinations of these three technologies to the connection of gate-type qubits is described.

## Results

### Connection qubit for scalability of two-dimensional implementations

**Figure 2:**
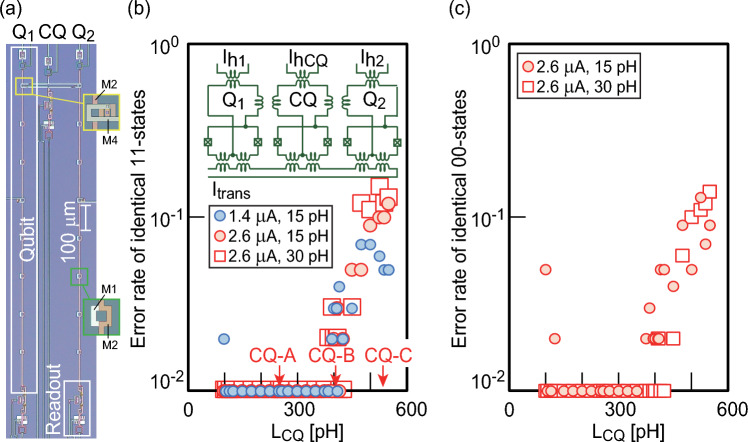
**(a)** Optical photograph of the superconducting quantum circuit. Q1 and Q2 are inductively coupled by the connection qubit (CQ). Each flux qubit has a readout for monitoring the individual qubit state. The insets of green and yellow regions show magnified views of the main loop of Q2 and the inductive coupling between Q1 and CQ, respectively. (**b)** Simulation results for the error rate of the identical 11 states with respect to *L*_CQ_. In the 11 state, both Q1 and Q2 are in the 1 state after sweeping the transverse magnetic flux through *I*_trans_. The *I*_c_ of each qubit and the *M* between the qubit and the CQ are considered as parameters. CQ-A (*L*_CQ_ = 258 pH), CQ-B (*L*_CQ_ = 408 pH), and CQ-C (*L*_CQ_ = 538 pH), which have different error rates, are fabricated. **c** Simulation results for error rates in the identical 00 states. There are no major differences between the simulations of the 11 and 00 states.

We examine the qubit interconnection by using the superconducting flux qubit (Fig. 2a). To confirm reliable coupling, a quantum circuit containing two flux qubits (Q1 and Q2) connected by the CQ is prepared. The qubit, coupled by the CQ, has the same design as the qubit used in our previous work^30,31^. The sample is fabricated by the superconducting multilayer process^32^. Q1, Q2, and CQ are constructed on the same chip. The main loop of the qubit is constructed using the superconducting wiring layers M1 to M4. Here, the CQ serves as the interconnection, as shown in Fig. 1a. Inductive coupling between Q1 (Q2) and CQ is achieved through superconducting rings constructed with multilayer wiring. The inset (yellow rectangle region) in Fig. 2a is a magnified view of the inductive coupling created by the overlapping superconducting rings. The ring diameter is 17.5 µm, the width is 5 µm, and the mutual inductance (*M*) is 15 pH. An appropriate design for the CQ, which ensures the correct qubit coupling after annealing, is investigated in a simulation. Herein, annealing means modulating a transverse magnetic flux applied to the qubit from 0 to $\Phi$_0_ using the current *I*_trans_; therefore, the shape of the energy potential in the qubit changes while the qubit remains in the ground state^14^. The shape of the energy potential varies with the amplitude of the total effective inductance (*L*). If the qubit interconnection is successful, then Q1 and Q2 take the same state (00 or 11). An error is defined as when the two qubits do not have identical states after annealing. We have calculated the frequency of identical state generation with 100 iterations for each inductance (*L*_CQ_) value. We define the error rate as the number of occurrences of nonidentical states divided by the number of iterations. An error rate of 10^−2^ means that no error occurred during the calculation. Errors are likely to occur when the *L*_CQ_ exceeds 400 pH (Fig. 2b). There is no difference in the threshold value of *L*_CQ_ for the error trend with respect to the critical current (*I*_c_) in the JJ or the magnitude of the mutual inductance (*M*) between the qubit and CQ. No major differences are observed between the error rates of identical 00 states and identical 11 states (Fig. 2c).

**Figure 3:**
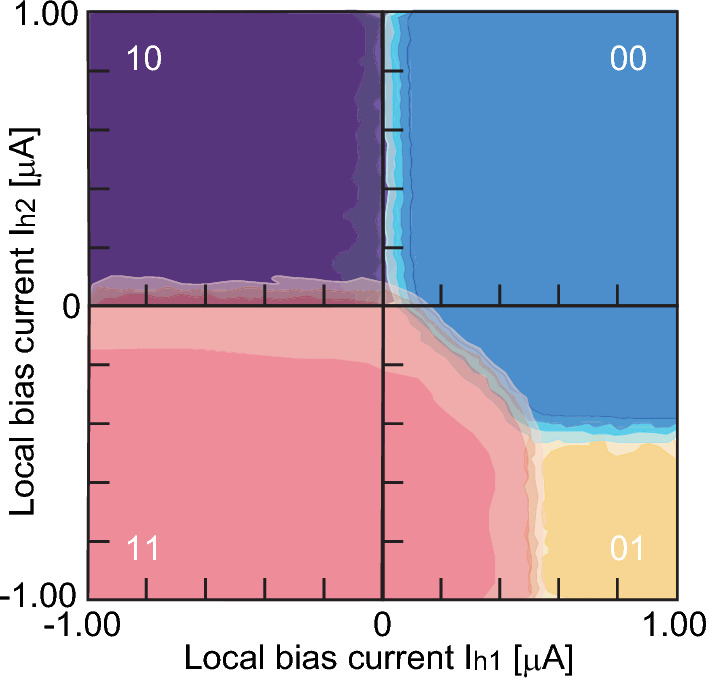
Phase diagram of qubit states expressed by Q1 and Q2 coupled by the connection qubit (CQ). Local bias currents (*I*_h_) are supplied to each flux qubit. The probabilities of each qubit state are evaluated experimentally under each bias condition with 10^4^ iterations at 10 mK. The shading represents the probability of each state from 1 to 0. The red and blue regions represent states in which the two flux qubits have identical states.

**Figure 4:**
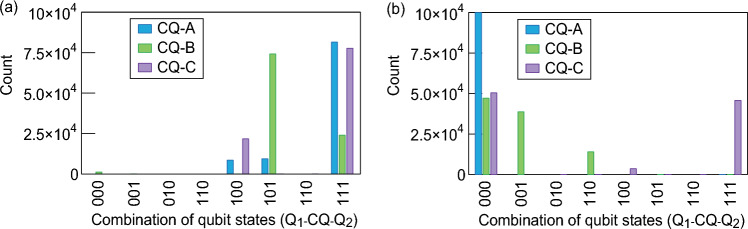
Combinations of the three qubit states after sweeping the transverse magnetic flux using the sample shown in Fig. 2a under conditions where Q1 is likely to be in (**a)** the 1 state (*I*_h1_ =  − 1 μA) or (**b)** the 0 state (*I*_h1_ = 1 μA). Here, *I*_h2_ and *I*_hCQ_ are both 0 μA. The experiment is conducted at 10 mK with 10^5^ iterations.

We examine the *L*_CQ_ values of 258 pH (CQ-A), 408 pH (CQ-B), and 538 pH (CQ-C) in Fig. 2b to show different trends in the error rates. We fabricate the superconducting quantum circuits in which the flux qubits Q1 and Q2 are connected by these three CQs. This corresponds to the superconducting quantum circuit having an interconnection between qubits on the same chip, as shown in Fig. 1a. We investigate the possible states of Q1 and Q2 in a 10-mK experiment. The phase diagram of two qubits connected by CQ-A shows a wide range of in-phase states (00, 11) after QA (Fig. 3). We focus on a region of the 00 state where both Q1 and Q2 are in the 0 state. The size of this region depends on the annealing time (*T*_a_). The gray zone, corresponding to a transition region, expands as *T*_a_ decreases (Supplementary Fig. 1 and Supplementary Note 1). *L*_CQ_ controls the region of the 00 state for CQ-B and CQ-C (Supplementary Fig. 2). Accordingly, the histograms showing the combinations of qubit states change (Fig. 4). The generation of the in-phase state of the 000 state (Q1-CQ-Q2) reaches 10^5^ counts, whereas the number of counts for the 111 state is smaller because of the flux from the peripheral circuit. The in-phase state is generated with high frequency in CQ-A; however, other states appear in CQ-B and CQ-C. This feature corresponds to the simulation result shown in Fig. 2. Applying a negative flux for the self-bias compensates for the offset flux from the circuit periphery, reducing errors in CQ-A (Supplementary Fig. 3). The frequency of the 000 states could not be improved by applying the flux for the self-bias in CQ-B and CQ-C (Supplementary Figs. 4 and 5). These results are related to the energy potential shape in the CQ and to whether a metastable state appears.

Conventional variable coupling has the constraint of a monostable energy potential^19^. This limits the magnitude of the *L* and, hence, the distance that can be connected by the variable coupler. Conversely, the CQ length modulates the qubit interconnection length. The CQ allows coupling between qubits with long distances of millimeters and with a strong coupling force; approximately 10 times stronger than conventional variable coupling^19^. Consequently, the CQ improves the two-dimensional scalability in QA. A factorization circuit is presented as an example of the use of interconnection by the CQ. By using a multiplier unit (MU) as a unit lattice connected by the CQ, factorization can be solved by QA^30^. The *n*-bit factorization circuit is implemented with 6(*n*/2)^2^ + *n*^2^ − 2*n* superconducting flux qubits. The 6-bit factorization circuit is constructed using 9 MU_*ij*_ (*i*, *j* = 0–2) and 24 CQs (Supplementary Fig. 6 and Supplementary Note 2). *L*_CQ_ is designed with a value of 241 ± 7 pH and *M* of 12 pH. A long interconnection exceeding 7 mm couples MU_02_ and MU_12_ (Supplementary Fig. 6c). Prime factorizations of integers of 15, 35, and 49 are simulated (Supplementary Fig. 7), demonstrating coupling through the CQ. The 4-bit factorization is confirmed experimentally through the QA circuit comprising interconnection using the CQ^33^.

### Precise control of coupling status in the CQ

**Figure 5:**
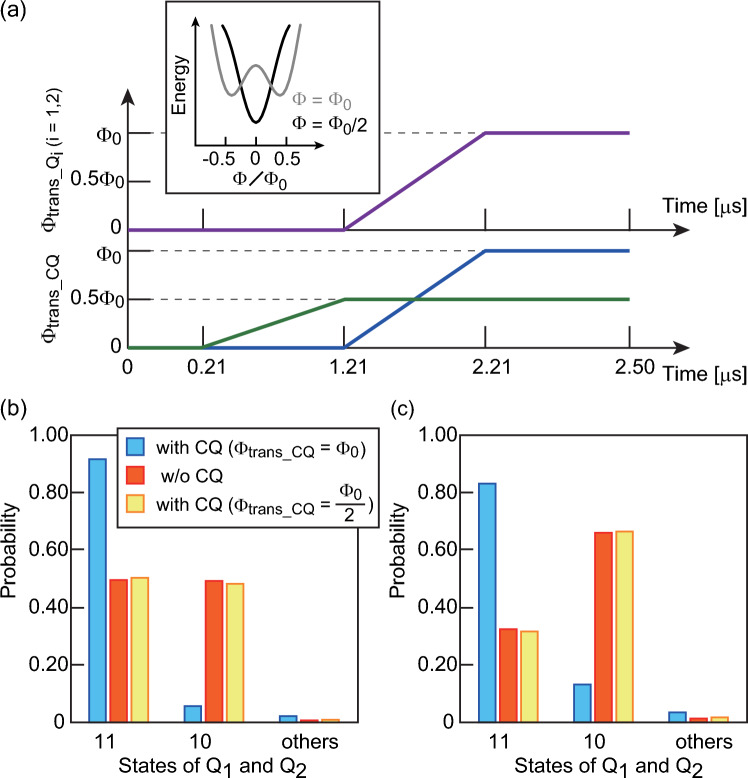
**(a)** Sequence of the flux applied to the qubit to control the coupling status. States of Q1 and Q2 are analyzed using $\Phi$_trans_Q*i*_ (*i* = 1,2) during 1.21–2.21 μs. When the coupling between Q1 and Q2 is set to the on state, the same time-dependent fluxes are applied to all qubits [with $\Phi$_trans_CQ_ = $\Phi$_0_ for the connection qubit (CQ)]. When the coupling is set to the off state, $\Phi$_trans_CQ_ of 0.5$\Phi$_0_ is applied to the CQ prior to Q1 and Q2 (with $\Phi$_trans_CQ_ = $\Phi$_0_/2 for the CQ). **(b)** The state probabilities of Q1 and Q2 with (*I*_h1_, *I*_h2_) = (1.2, 0) μA. **c** The state probabilities of Q1 and Q2 with (*I*_h1_, *I*_h2_) = (1.2, − 0.2) μA. For comparison, a model with no interactions between Q1 and Q2 is also calculated (w/o CQ). Each probability is evaluated from the simulation results with 10^3^ iterations.

Interconnection requires the precise controllability of the coupling status between qubits by applying a flux to the CQ. Simulations are performed to investigate the coupling status between two qubits under various fluxing conditions. The transverse magnetic fluxes of $\Phi$_trans_Q*i*_ (purple line) and $\Phi$_trans_CQ_ (blue and green lines) (Fig. 5a) are applied to each qubit and the CQ. Because inductive coupling via the CQ always works, it is necessary to devise methods to turn off the coupling status to avoid unintentional crosstalk. Here, the probabilities for various combinations of states between Q1 and Q2 are calculated with 1000 iterations. We consider the local bias current condition in which Q1 and Q2 adopt the 1 state with probabilities of 100% and 50%, respectively, without interactions between Q1 and Q2. This corresponds to Q1 and Q2 being isolated, resulting in 11 states with a probability of 50% (w/o CQ in Fig. 5b). In contrast, 11 states mainly appear when the transverse magnetic fluxes are applied with modulation from 0 to $\Phi$_0_ for all qubits (CQ with $\Phi$_trans_CQ_ = $\Phi$_0_, described with the blue line in Fig. 5a). This means that the qubit coupling is turned on. This mechanism is used for the prime factorization in the 6-bit factorization circuit. Next, the CQ is annealed with $\Phi$_trans_CQ_ of 0.5$\Phi$_0_ before the annealing of Q1 and Q2 (described with the green line in Fig. 5a). In this case, the CQ energy potential has a single-well shape during the annealing of Q1 and Q2, and the 11 and 10 states appear with half the probability (CQ with $\Phi$_trans_CQ_ = $\Phi$_0_/2 in Fig. 5b). This result is the same as those when Q1 and Q2 are isolated, meaning that the qubit interconnection is turned off. Another bias condition, in which Q1 and Q2 are predominantly in the 1 and 0 states, respectively, is also considered for each qubit being isolated (Fig. 5c). Even under this bias condition, the 11 state mainly appears after annealing with the transverse magnetic fluxes modulated from 0 to $\Phi$_0_. If the CQ is preannealed with $\Phi$_trans_CQ_ of 0.5$\Phi$_0_, the qubits take the same states that Q1 and Q2 are independently present. There is a slight interaction between qubits even if the flux is not applied to the CQ because of inductive coupling (Supplementary Figs. 8 and 9 and Supplementary Note 3). It is important to change the time variation of the energy potential between the CQ and the coupled qubits (Q1 and Q2), which eliminates quantum tunneling between the two qubits and turns off the coupling of unused unit lattices in the quantum circuits, avoiding unintentional crosstalk. To have Q1 and Q2 in the same state with more than 90% probability, more than 50 pH of *M* is required when the qubits are coupled with superconducting wires with the same value of *L* in the CQ. In contrast, the CQ achieves strong coupling between qubits through overlapping the rings with an *M* of only 15 pH, as shown in Fig. 2a. Note that the CQ can precisely control the coupling status between the on and off states.

### Flip-chip bonding for the 2.5-dimensional implementation

**Figure 6:**
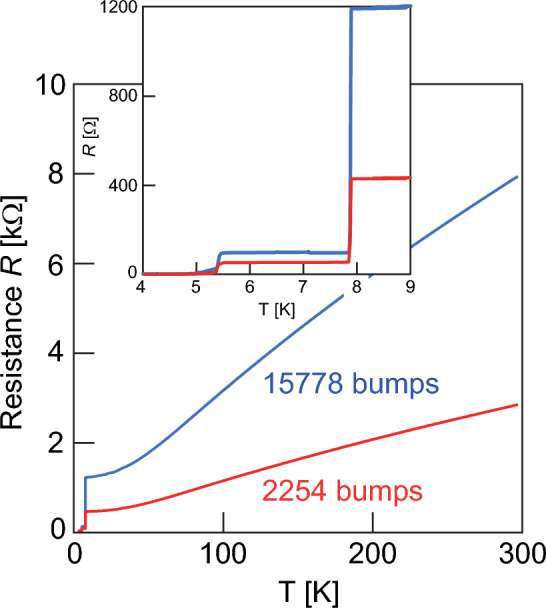
Temperature dependence of the resistance of a circuit composed of Nb wires connected by In–Sn bumps. The Nb wiring circuits were configured in series with 2254 (red line) and 15,778 (blue line) solder bumps, respectively.

Further scalability can be achieved by developing the routing to provide 2.5-dimensional interconnection with the CQ. Here, we examine the flip-chip bonding method required to extend the coupling scheme between qubits as shown in Fig. 1b. A Nb wiring circuit connected in series with solder bumps (daisy-chain structure) is fabricated by flip-chip bonding to verify the superconducting connections. Daisy chains are constructed by spreading over the entire 5-mm square chip. The total length of the daisy-chain is 45 mm for 2254 bumps and 315 mm for 15,778 bumps. Bumps have a diameter of 20 µm and a pitch interval of 20 µm. The daisy-chain Nb wire sample is mounted on a refrigerator and cooled through the bottom chip. Figure 6 shows the resistance–temperature characteristics. The Nb wire undergoes a superconducting transition at approximately 8.0 K. The plateau in the resistance from 5.4 to 7.9 K is due to the bumps. Although the effect of contact resistance is included, dividing this value by the number of bumps gives the resistance per unit bump. The resistance is suppressed at approximately 5 K, where the bumps undergo the superconducting transition. This result indicates that the upper Nb wire is cooled via the bumps, resulting in the whole circuit exhibiting superconducting behavior. The confirmation of the continuity with the daisy-chain configured by spreading over the entire chip suggests the possibility of the outer-connection between 5-mm-square chips as shown in Fig. 1b.

**Figure 7:**
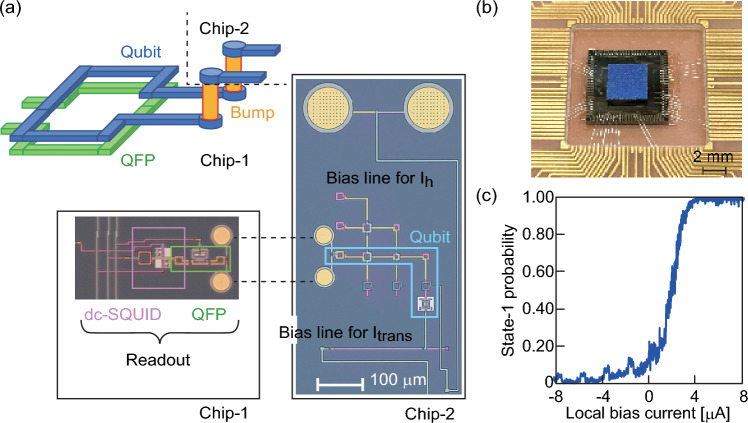
**(a)** Schematic (perspective view) of 2.5-dimensional flux qubit using flip-chip bonding for coupling between a quantum flux parametron (QFP)^16^ and a qubit [top], and photographs (top view) of chip-1 and chip-2 [bottom]. The flux qubit is split across the two chips, with bumps providing connectivity between the two sections. The main loop in the qubit and a bias line for *I*_h_ are constructed in 2.5-dimensions by flip-chip bonding. (**b)** Photograph of the 2.5-dimensional quantum circuit. The top and bottom chips correspond to chip-1 and chip-2.** c** State-transition probability in the 2.5-dimensional flux qubit measured experimentally at 4.2 K. The probability is evaluated under each current condition with 10^3^ iterations.

We fabricate the readout circuit coupled with a part of the CQ in Chip-1 (Fig. 7a). Main component of the CQ is fabricated in Chip-2. Next, Ti/Au adhesive regions are formed on the Nb electrodes, and then In–Sn bumps are formed on the Ti/Au regions by reflow soldering (Supplementary Note 4 and Supplementary Figs. 10 and 11). The bump diameter varies near the circuit fine pattern and in the peripheral area, allowing heat conduction between chips when cooling in the refrigerator and reducing the bump area. To avoid changes in the characteristics of the JJ, the bonding is performed below 150 °C^30^. Part of the main loop of the CQ and the control line of the local bias current are on the Chip-1 side, and these structures are completed by flip-chip bonding. The fabrication process is similar for the daisy-chain Nb wire sample and the sample shown in Fig. 7b. Figure 7b shows an optical micrograph of two chips connected via bumps. The CQ has the same state transition as the conventional qubit in the experiment at 4.2 K (Fig. 7c). The entire circuit connected through the bumps is in the superconducting state, resulting in the qubit operation. To our best knowledge, this is the first demonstration of qubit operation by forming the main loop with a flip-chip connection, which enables 2.5-dimensional circuit routing.

### Novel interconnection using the superconducting flux qubit

A novel interconnection is realized by combining the three technologies described in previous sections. We propose an idea for the outer-connection using the CQ, which couples qubits fabricated on different chips as shown in Fig. 1b. The coupling status of two qubits can be switched by controlling the shape of the CQ energy potential over time. This would allow us to increase the number of qubits by connecting chips where the characteristics of the qubits are uniformly constructed through fabrication.

**Figure 8:**
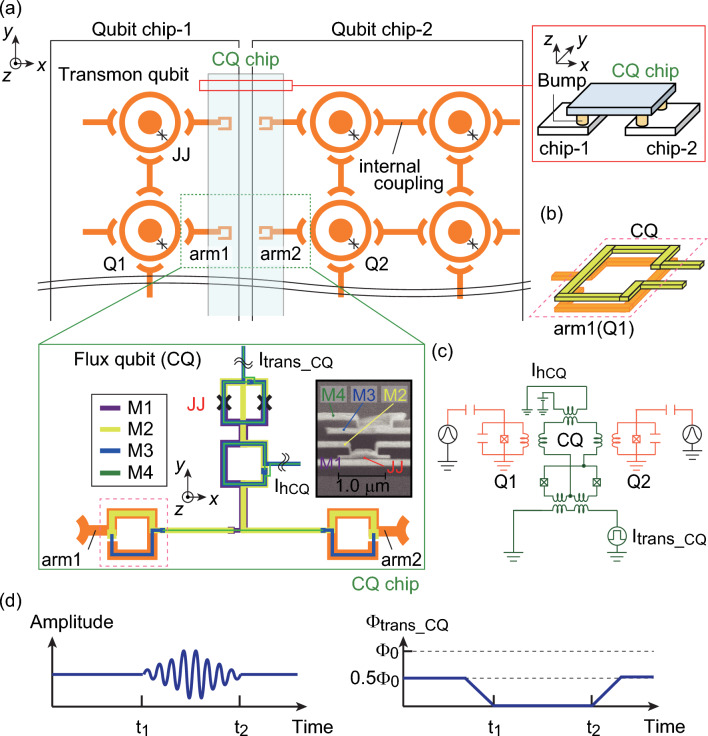
(**a**) Layout of interconnection between transmon qubits and the connection qubit (CQ) in different chips. The chips are fixed by flip-chip bonding in the light-blue regions. The CQ indirectly couples the transmon qubits at the edges of the chips. The coupling is achieved by overlapping the area between part of the transmon qubit and part of the main loop consisting of M2 and M3 layers in the CQ. **(b)** Schematic of the interconnection between the transmon qubit and CQ. (**c)** Equivalent circuit model. The interconnection is controlled by applying the transverse magnetic flux to the CQ through the current *I*_trans_CQ_. (**d)** Signal sequence during the gate operation. During the off state, the energy potential of the CQ is controlled to be a single-well potential.

We expect that this technology can be used for interconnection in gate-type qubits. Chen et al.^34^ reported an interconnection method in which two qubits were coupled using a variable JJ coupler. Controlling the coupling status with this coupler transferred a long-distance quantum state between two qubits combined with a superconducting coaxial cable^35^. The JJ^34^, a superconducting quantum interference device (SQUID)^36^, and a resonator^37^ are used to realize the coupling switch function. If the detuning of the coupler and the qubit is large, then mostly single transmission is achieved^35^. The switching is controlled by applying a flux. In contrast, we consider a scaling method with 2.5-dimensional connections between the chips. We assume that there are three qubit chips (Fig. 8a): two base chips for the gate-type qubits (Qubit chip-1 and Qubit chip-2) and a top chip for the CQ (CQ chip). Parts of the transmon qubit and the CQ face each other to create indirect coupling (Fig. 8b). The ground planes are electrically connected via bumps fabricated by flip-chip bonding. Figure 8c shows the equivalent circuit of the three qubits. The interconnection is controlled by applying a transverse magnetic flux to the CQ through *I*_trans_CQ_ (Fig. 8d). To turn the coupling off, a flux of 0.5$\Phi$_0_ is applied to the CQ (0 − *t*_1_, *t* > *t*_2_). During the gate operation, the coupling is turned on by not applying the flux to the CQ (*t*_1_–*t*_2_). The possibility of interaction between Q1 and Q2 via the CQ is considered with the proof-of-concept simulation (Supplementary Note 5 and Supplementary Fig. 12).

## Discussion

The internal long-distance connection of qubits in the same chip is possible with the CQ. Toward the outer-interconnection, construction of a CQ with components in different chips is expected to be realized using flip-chip bonding. The confirmation of superconductivity in the daisy-chain in the 5-mm-square chip suggests the possibility of the outer-connection between chips as shown in Fig. 1b. In our future work, implementation of the outer-connection between different chips and controlling the coupling status by modulating the CQ flux will be demonstrated simultaneously. In the gate-based architecture, we should consider that the long-distance connection would have parasitic capacitance, which has been neglected in this analysis. The dynamics of the operation and loss mechanisms are also critical issues. A detailed analysis with these components will be investigated in future work. Practically, there are two problems. The first challenge is to prevent dielectric loss in the gate-type qubits because the CQ is composed of an insulator with a superconducting multilayer. The dielectric should not be close to the gate-type qubit. One feasible way to achieve this is by restricting the overlap of the gate-type qubit and CQ chips around the coupling area. Providing some ground-connected dummy bumps would be effective because they cover the coupling area with a ground potential. The second challenge is the short coherence time in the interconnection. The CQ coherence time is as short as several tens of nanoseconds because of the dielectric loss. During gate operation, the time that coupling can be turned on must not be limited. Changing the interlayer dielectric from SiO_2_ to SiN^38^ should increase the coherence time. We expect that solving these problems will allow the CQ to pave the way for new quantum circuits that use combinations of several types of qubits.

## Methods

### Fabrication of the superconducting flux qubit

The superconducting quantum circuit is patterned on a 3-in. diameter high-resistivity (> 1000 $\Omega$cm) silicon substrate. The circuit is fabricated by a process that creates four Nb layers and JJs with a critical current density of 1 μA/μm^2^. Each Nb wiring layer is spaced by a SiO_2_ insulating film, with M1, M2, M3, and M4 superconducting film layers built on the silicon substrate^32^. The JJ is stably formed over a 1 × 1 μm square. In our work, the JJ for the superconducting flux qubit is a 2.5 × 2.5-μm square. The value of *L* is rigorously designed using InductEX^39^. The consistency of the simulated and experimental values of *L* is confirmed. The values of *L* and *I*_c_ of the qubit are 299 pH and 2.5 μA, respectively. Additional fabrication details are available in Refs.^30–32^.

### Flip-chip integration

The Ti/Au pattern is formed by sputtering and lift-off where the bump is constructed later. Bumps of approximately 10 μm in height are formed only on the Ti/Au patterns when the chip is immersed in a molten In–Sn alloy in a solder bath. A flip-chip bonder is used for pressing two chips together. Bonding is performed below 150℃ with a typical bonding force of 10–20 N in the bump area, which results in a compression of approximately 10 μm in the total height of the two solder bump depositions. In our method, the bottom chip (readout) can be used with different flip-chip bonding configurations by changing the circuits on the top chip (qubit), enabling circuits with various functions to be realized (Supplementary Note 4 and Supplementary Fig. 11).

### Measurement

The measurements in Figs. 3 and 4 and Supplementary Figs. 1–5 are taken using our standard measurement techniques^21,31^ with a commercial dilution refrigerator (XLD250, BlueFors) operating at a base temperature of approximately 10 mK. The measurement in Fig. 7 is taken using our standard measurement with a helium dewar at 4.2 K. The measurement setup is at room temperature. The signals from the readout circuit are monitored by an oscilloscope after the synchronizing transverse magnetic fluxes are applied to the three flux qubits. *T*_a_ is controlled by the rise time of the current, which produces the flux. Typically, a rise time of 100 μs is used. After QA, the qubit has two bistable states with persistent current flowing clockwise or counterclockwise. We define these two states so that they correspond to logical 1 and 0 states. Additional measurement details are available in Refs.^30,33^.

### Simulation

The analyses in Figs. 2 and 5 and Supplementary Figs. 7–9 and 12 are conducted using the Josephson integrated circuit simulator^40^. The qubit, readout circuit, and inductive coupling are expressed by the equivalent circuit using the circuit parameter. When a noise current is used, the simulation corresponds to the experimental results^31^. Additional simulation details are available in Refs.^21,30^.

### Supplementary Information

Supplementary Information.

## Data Availability

All data generated or analyzed during this study are included in this published article (and its Supplementary Information files).
