# Supplementary material for: Scalable interconnection using a superconducting flux qubit
Source: Sci Rep. 2024 Jul 16;14:16447. doi: 10.1038/s41598-024-65086-1 (PMC11252359; doi:10.1038/s41598-024-65086-1)
Supplement: Supplementary file 1 — Supplementary Information. [file 41598_2024_65086_MOESM1_ESM.pdf]

Supplementary Information for

Scalable Interconnection Using a Superconducting Flux Qubit

Daisuke Saida[1,2*], Kazumasa Makise[2,3] and Mutsuo Hidaka[2]

[1]Fujitsu Limited, 1-1, Kamikodanaka 4-chome, Nakahara-ku, Kawasaki, Kanagawa, 211-8588, Japan

[2]National Institute of Advanced Industrial Science and Technology, Ibaraki, Japan

[3]National Astronomical Observatory of Japan, Tokyo, Japan

*Corresponding author: saida.daisuke@fujitsu.com

Supplementary Note 1: Characteristics of the phase diagram in two qubits coupled by the CQ

We focus on the region where both qubits (Q1 and Q2) take the 0 state (00 state) in Fig. 3 and discuss its characteristics. Supplementary Figs. 1**a**, 1**b**, and 1**c** show the phase diagram of the 00 state in CQ-A for annealing times (T_a) of 100, 15, and 1 μs, respectively. For simplicity, only the 00 state is shown. The gray zone, which is the transition area between states, increases as T_a decreases. The experiment is mainly carried out at T_a of 100 μs because the in-phase state of two qubits can be observed over a wide range of current conditions. This is also related to the mechanical design of wires through which the voltage that provides the magnetic flux passes. The whole experimental system is designed to propagate voltage signals from several kilohertz to 100 kHz.

Supplementary Figs. 2**a**, 2**b**, and 2**c** show the phase diagrams of the 00 states in CQ-A, CQ-B, and CQ-C, respectively, at T_a of 100 μs. The local bias current (I_h) condition that gives the 00 state becomes narrower as the value of L in the CQ (L_{CQ}) increases. We focus on different features in the phase diagram of qubits coupled by CQ-B and CQ-C. When the state transition probability of CQ-C is evaluated alone, it shows no monotonic change with I_h. This is mainly due to the shape of the energy potential in the CQ[2]. The metastable state in the energy potential begins to appear above β_L of 8. In CQ-B, the value of β_L is designed to be about 6. Although no metastable state appears in the energy potential, the designed conditions for the qubit are in the boundary where the qubit state does not change monotonically with the flux. Supplementary Figs. 3**a**, 3**b**, and 3**c** show histograms of the combination of qubit states (Q1-CQ-Q2) at T_a of 100, 15, and 1 μs, respectively, for qubits coupled with CQ-A. The 000 and 111 states occur frequently. Supplementary Figs. 4**a**, 4**b**, and 4**c** show histograms of the combination of qubit states (Q1-CQ-Q2) at T_a of 100, 15, and 1 μs, respectively, for qubits coupled with CQ-B. The 110 state occurs frequently in conditions with I_{h1} of 1.0 μA. This means that the 10 region is extended in the phase diagrams of Q1 and Q2. Supplementary Figs. 5**a**, 5**b**, and 5**c** show histograms of the combination of qubit states (Q1-CQ-Q2) at T_a of 100, 15, and 1 μs, respectively, for qubits coupled with CQ-C. Similar to the results for CQ-B, the 110 states occur frequently in conditions with I_{h1} of 1.0 μA. The 111 state also appears with I_{h1} of 1.0 μA, meaning that the 11 region is extended in the

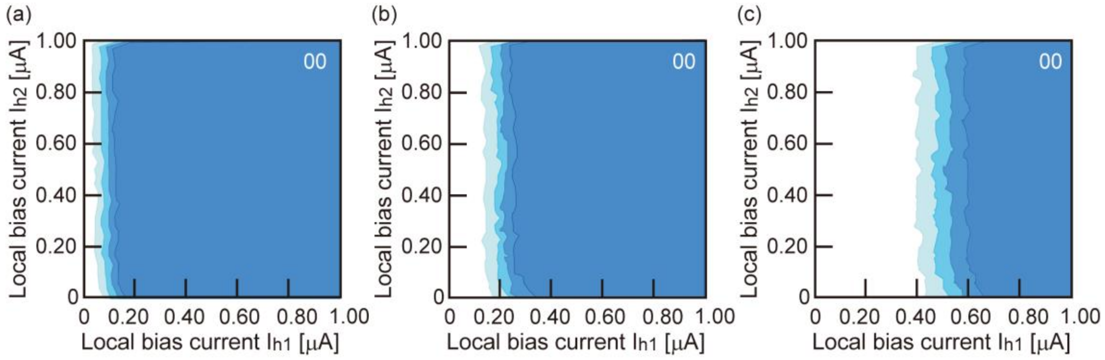

Supplementary Fig. 1 Phase diagram of 00 state with T_a of **a** 100, **b** 15, and **c** 1 μs. The experiment is carried out at 10 mK using CQ-A (L_{CQ} = 258 pH). To clarify the T_a dependence of the 00 state, other regions are not shown. The color shading represents the probability from 0 to 1 that the 00 state occurs.

phase diagrams of Q1 and Q2. The metastable energy potential of CQ-C may affect these features. In Fig. 4, the generation of the 000 and 111 states cannot be intentionally controlled by using CQ-B and CQ-C. The reason for this is related to the distribution of the identical states in the phase diagram. In QA, the CQ should be designed with β_L from 1.2 to 6 so that the energy potential adopts a clear double-well shape when transverse flux (Φ_{trans_CQ}) of Φ_0 is applied.

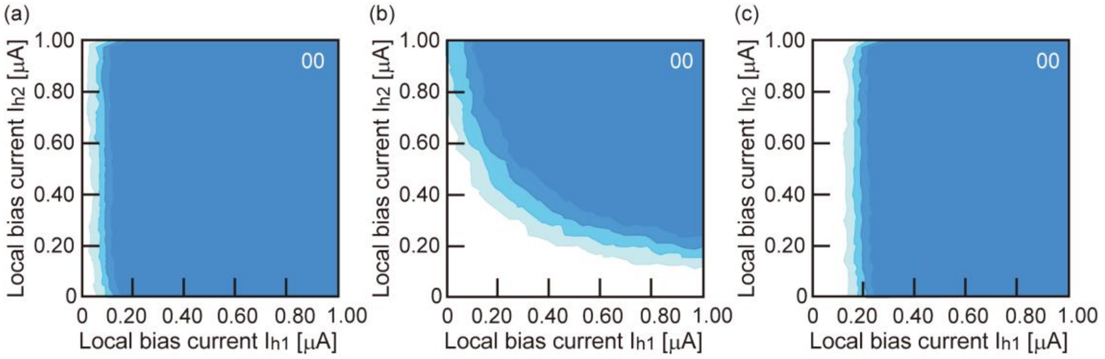

Supplementary Fig. 2 Phase diagram of 00 state in qubits coupled experimentally by **a** CQ-A (L_{CQ} = 258 pH), **b** CQ-B (L_{CQ} = 408 pH), and **c** CQ-C (L_{CQ} = 538 pH) with T_a of 100 μs at 10 mK. To clarify the L_{CQ} dependence of the 00 state, other regions are not shown. The color shading represents the probability from 0 to 1 that the 00 state occurs. The β_L values of CQ-A, CQ-B, and CQ-C are 3.9, 6.2, and 8.2, respectively. Above β_L of 6.0, the energy potential does not change monotonically with the flux[1].

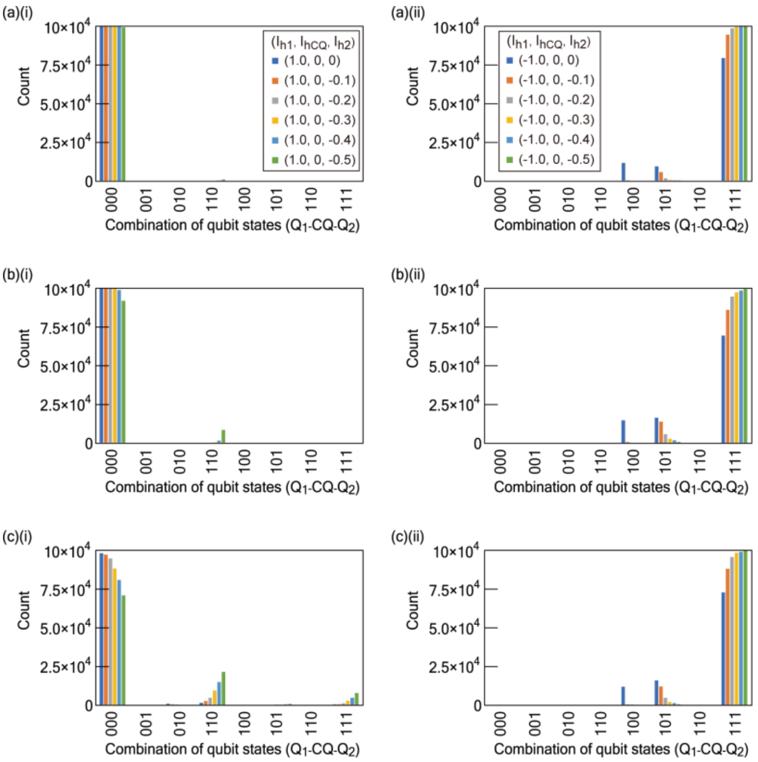

Supplementary Fig. 3 Histograms of each qubit state in two qubits coupled by CQ-A after QA with T_a of 100 μs under current conditions of **a(i)** I_{h1} = 1.0 μA and **a(ii)** I_{h1} = -1.0 μA, with T_a of 15 μs under current conditions of **b(i)** I_{h1} = 1.0 μA and **b(ii)** I_{h1} = -1.0 μA, and with T_a of 1 μs under current conditions of **c(i)** I_{h1} = 1.0 μA and **c(ii)** I_{h1} = -1.0 μA. Experiments are carried out at 10 mK. In each experiment, the local bias current of CQ (I_{hCQ}) is kept at 0 μA. The local bias current of Q2 (I_{h2}) is modulated from -0.5 to 0 μA.

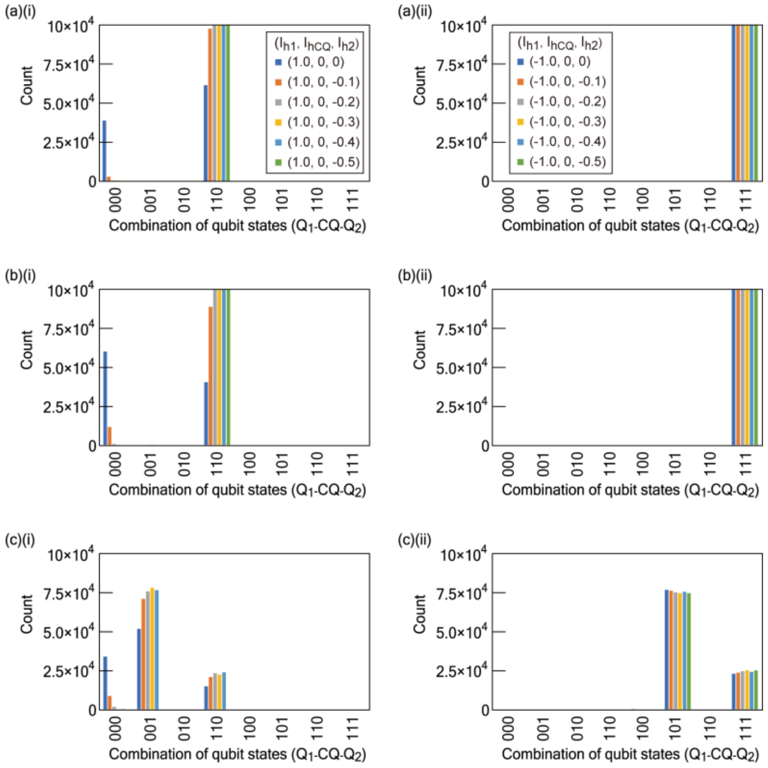

Supplementary Fig. 4 Histograms of each qubit state in two qubits coupled by CQ-B after QA with T_a of 100 μs under current conditions of **a(i)** I_{h1} = 1.0 μA and **a(ii)** I_{h1} = -1.0 μA, with T_a of 15 μs under current conditions of **b(i)** I_{h1} = 1.0 μA and **b(ii)** I_{h1} = -1.0 μA, and with T_a of 1 μs under current conditions of **c(i)** I_{h1} = 1.0 μA and **c(ii)** I_{h1} = -1.0 μA. Experiments are carried out at 10 mK. In each experiment, I_{hCQ} is kept at 0 μA. I_{h2} is modulated from -0.5 to 0 μA.

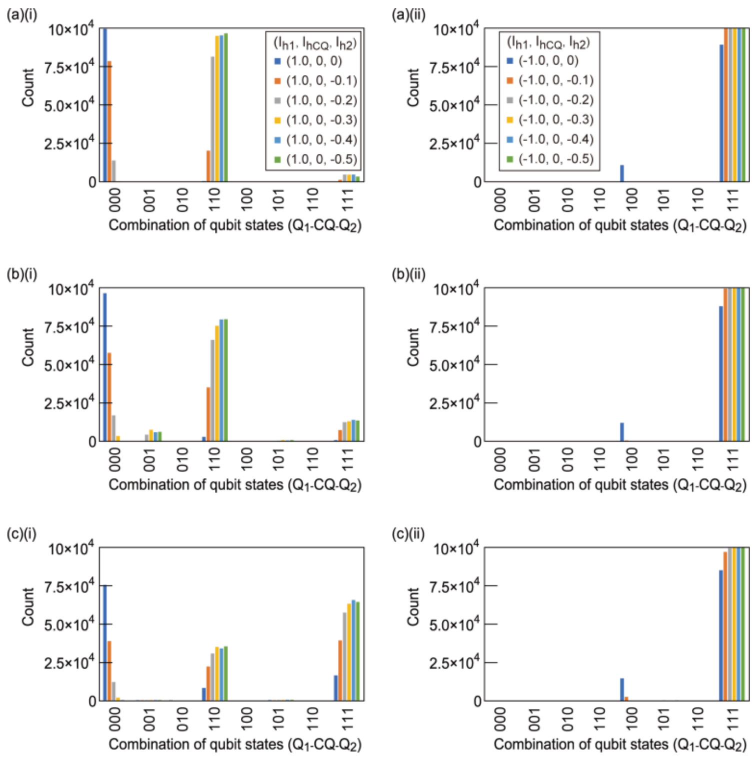

Supplementary Fig. 5 Histograms of each qubit state in two qubits coupled by CQ-C after QA with T_a of 100 μs under current conditions of **a(i)** I_{h1} = 1.0 μA and **a(ii)** I_{h1} = -1.0 μA, with T_a of 15 μs under current conditions of **b(i)** I_{h1} = 1.0 μA and **b(ii)** I_{h1} = -1.0 μA, and with T_a of 1 μs under current conditions of **c(i)** I_{h1} = 1.0 μA and **c(ii)** I_{h1} = -1.0 μA. Experiments are carried out at 10 mK. In each experiment, I_{hCQ} is kept at 0 μA. I_{h2} is modulated from -0.5 to 0 μA.

Supplementary Note 2: 6-bit factorization circuit

In quantum annealing (QA), we design a problem Hamiltonian with the ground state corresponding to the solution. The desired solution can be obtained by giving a local bias current for initial conditions, and invertible logic[2] can also be used. We can perform the prime factorization in QA based on invertible logic by using a classical multiplier Hamiltonian. Supplementary Fig. 6**a** shows a schematic of the classical 6-bit multiplier circuit. In the multiplication, inputs correspond to the combination of X = (X3, X2, X1) and Y = (Y3, Y2, Y1). Output is P = (P6, P5, P4, P3, P2, P1). Using the ground-state spin logic[3], the Hamiltonian of this circuit can be expressed as shown in Supplementary Fig. 6**b**. This Hamiltonian consists of the multiplier unit (MU) and qubit interconnections corresponding to carry propagation path shown in Supplementary Fig. 6**a**. The interconnection is realized with the CQ. The distances required for each interconnection are different, unlike the couplings between unit lattices in the chimera graph architecture[4].

Supplementary Fig. 6**c** is an optical micrograph of a 6-bit factorization circuit composed of 9 MUs and 24 CQs. The CQ connecting the longest distance is shown in red. The main loop of the CQ is configured so that the outward and return paths are opposite each other. The length of the coupling between the qubits exceeds 7 mm. As discussed in Supplementary Note 1, the design of L_{CQ} is important to prevent errors in the interconnection. The values of L_{CQ} are designed as an average of 241 pH with a standard deviation of ±7% (Supplementary Fig. 6**d**). Because each CQ has a different main loop length, we adjust both the loop width and use the metal layer to construct the loop. The 6-bit factorization circuit consists of 78 qubits, and 288 qubits are required if we directly implement the Hamiltonian shown in Supplementary Fig. 6**b** in the chimera graph architecture. Our method, in which the design of the superconducting quantum circuit directly corresponds to the Hamiltonian, can help to reduce the number of qubits needed, moving closer to practical use of QA.

Supplementary Figs. 7**a**, 7**b**, and 7**c** show the prime factorization of integers 15, 35, and 49, respectively, in simulations. In this analysis, the annealing is performed with the modulation of the transverse magnetic flux from 0 to Φ_0 during 0.02–1.02 μs. If the signal in the readout has a finite voltage (>10 μV) around 1.06 μs, the qubit takes the 1 state. $V(\mathrm{X}_{02})$, $V(\mathrm{X}_{01})$, and $V(\mathrm{X}_{00})$ correspond to X components X3, X2, and X1, respectively. $V(\mathrm{Y}_{22})$, $V(\mathrm{Y}_{12})$, and $V(\mathrm{Y}_{02})$ correspond to Y components Y3, Y2, and Y1, respectively. The prime factors are obtained for each integer. For example, in the prime factorization of integer 15, combinations of $X = (101)_{(2)}$ and $Y = (011)_{(2)}$ or vice versa are obtained. This proves that the CQ combines the unit cell properly to express the entire Hamiltonian.

The CQ enables the quantum circuit to be extended in two dimensions by combining the unit lattice. Because the connection distance varies for each CQ, the individual design of L_{CQ} is necessary. In this study, L_{CQ} was designed with InductEX[5] for the proof-of-concept demonstration; however, design automation is necessary to increase scalability. In semiconductor circuits, automated design is used for routing the wiring. A similar approach would be suitable for the layout of quantum circuits. In future work, automated design could be treated as a constrained problem with wire width as a parameter, or machine learning could be used.

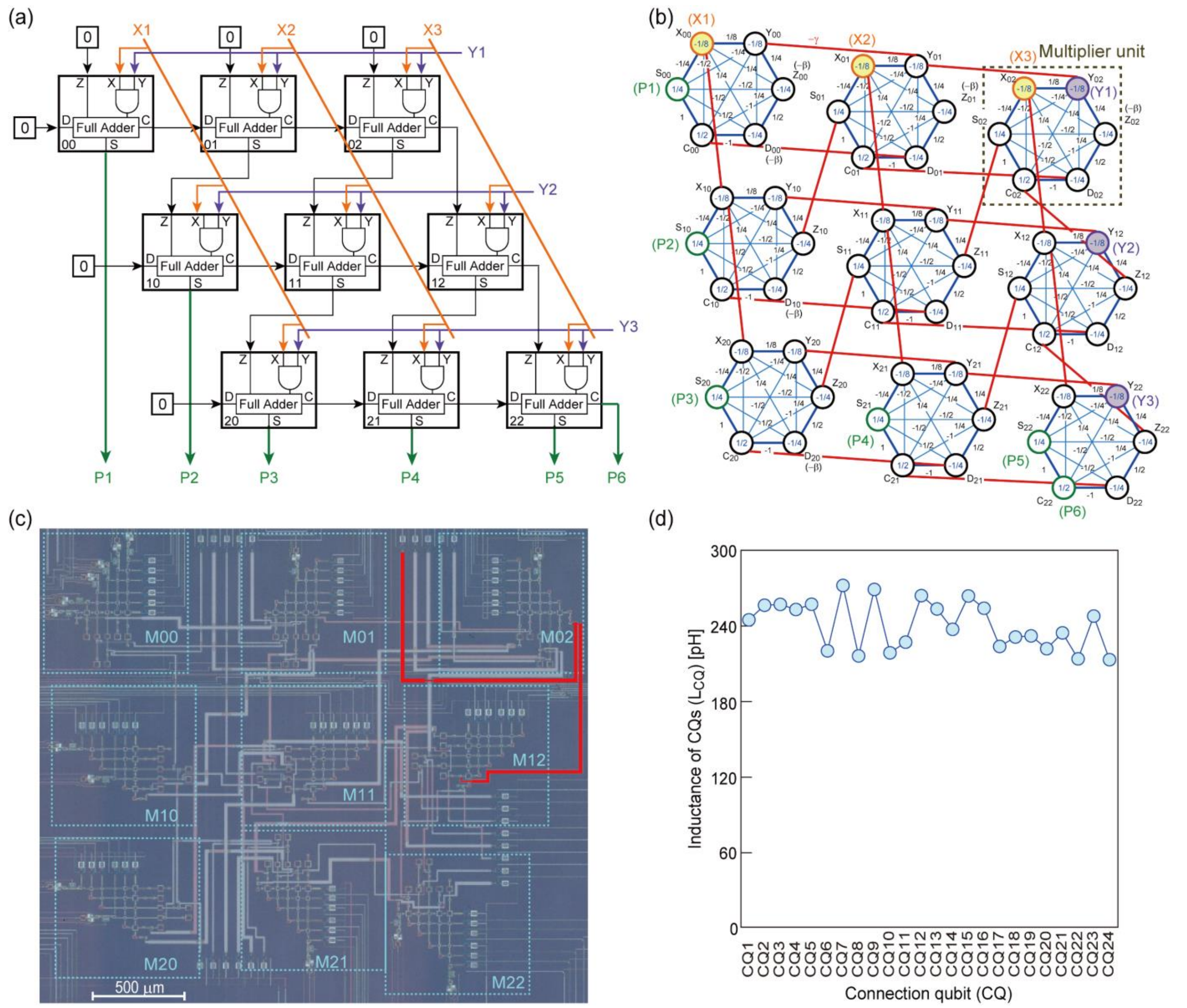

Supplementary Fig. 6 a Schematic of the classical 6-bit multiplier based on MUs (MU_{ij}, i, j = 0–2). **b** Description of the Hamiltonian in the 6-bit factorization circuit. Production can be described as $P = (P_6\ P_5\ P_4\ P_3\ P_2\ P_1)_{(2)} = (C_{22}\ S_{22}\ S_{21}\ S_{20}\ S_{10}\ S_{00})_{(2)}$. Inputs are represented as $X = (X_3\ X_2\ X_1)_{(2)} = (X_{02}\ X_{01}\ X_{00})_{(2)}$ and $Y = (Y_3\ Y_2\ Y_1)_{(2)} = (Y_{22}\ Y_{12}\ Y_{02})_{(2)}$, respectively. **c** Optical photograph of a superconducting quantum circuit embedding the 6-bit factorization Hamiltonian. The circuit is composed of 9 MUs and 24 CQs, and consequently uses 78 superconducting flux qubits. **d** Designed L_{CQ} values of the 24 CQs used in the 6-bit factorization circuit within the range of 241 ± 7 pH.

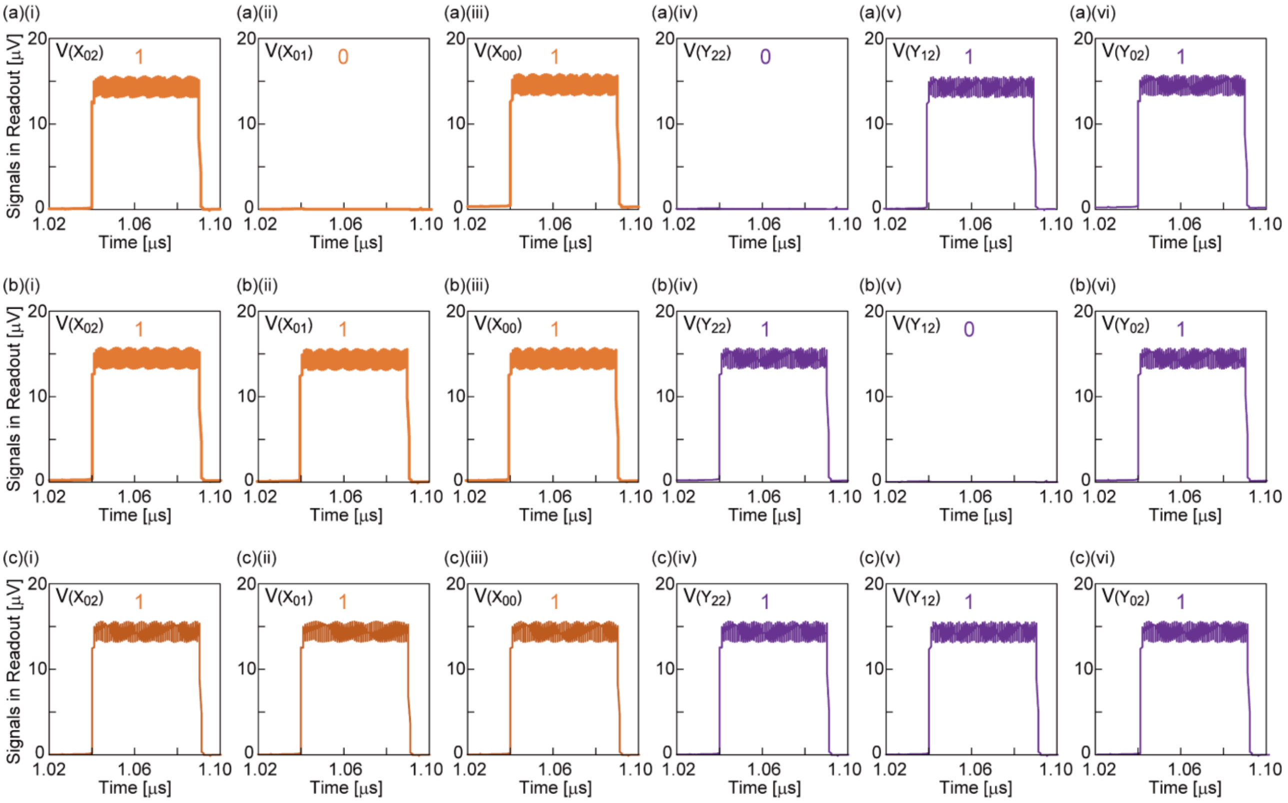

Supplementary Fig. 7 Output signals in readouts for prime factorization of integers P of **a** 15, **b** 35, and **c** 49 in JSIM analysis. Prime factor X is expressed in **(i)** X_3, **(ii)** X_2, and **(iii)** X_1. Prime factor Y is expressed in **(iv)** Y_3, **(v)** Y_2, and **(vi)** Y_1.

Supplementary Note 3: Turning off the qubit coupling via the CQ

We consider a model of two qubits (Q1 and Q2) coupled by the CQ. The transverse magnetic fluxes (Φ_{trans_i}, i = 1, 2) are applied to Q1 and Q2 with modulation from 0 to Φ_0 over 1.21–2.21 µs (Supplementary Fig. 8**a**). Five ways of applying the transverse magnetic flux ($\Phi_{\mathrm{trans\_CQ}}$) to the CQ are investigated. In the first, $\Phi_{\mathrm{trans\_CQ}}$ is 0 during the sweeping of $\Phi_{\mathrm{trans}i(i = 1,2)}$ in Q1 and Q2 (Supplementary Fig. 8**b(i)**). This is equivalent to no flux being applied to the CQ connecting the unit lattice that is not used. In the second and third ways, $\Phi_{\mathrm{trans\_CQ}}$ is modulated from 0 to 0.5Φ_0. The two ways differ in whether the $\Phi_{\mathrm{trans\_CQ}}$ is applied at the same time (Supplementary Fig. 8**b(ii)**) or earlier than Φ_{trans_i} to Q1 and Q2 (Supplementary Fig. 9**b(iii)**). In the fourth way, $\Phi_{\mathrm{trans\_CQ}}$ is modulated from Φ_0 to 0.5Φ_0 and is applied to the CQ at the same time as Φ_{trans_i} on the time axis (Supplementary Fig. 8**b(iv)**). In the fifth way, $\Phi_{\mathrm{trans\_CQ}}$ is modulated from Φ_0 to 0 and is applied to the CQ at the same time as Φ_{trans_i} on the time axis (Supplementary Fig. 8**b(v)**). To favor Q1 adopting the 1 state, the flux is applied through a local bias current (I_{h1}) of 1.2 µA. Calculations are performed under two current conditions for Q2. We use two I_{h2} values for which Q2 takes the 0 state with a probability of 50% (Supplementary Fig. 8**c**) and 70% (Supplementary Fig. 8**d**) when Q2 is isolated. The condition "w/o coupling" is based on the calculation results when Q1 and Q2 are isolated. Strictly off-state coupling between qubits means that the probability is the same as the condition "w/o coupling". The probability is different between conditions "0→0" (corresponding to constant $\Phi_{\mathrm{trans\_CQ}}$ of 0) and "w/o coupling", indicating that crosstalk occurs via the CQ. The coupling is strictly in the off-state when $\Phi_{\mathrm{trans\_CQ}}$ is varied from 0 to 0.5Φ_0 before applying Φ_{trans_i} to Q1 and Q2 ("0→0.5Φ_0 (forward)" in Supplementary Fig. 9). The energy potential of the CQ becomes a single well, existing at the lowest energy when the $\Phi_{\mathrm{trans\_CQ}}$ of 0.5 Φ_0 is applied. When $\Phi_{\mathrm{trans\_CQ}}$ is modulated from 0 to 0.5Φ_0, the coupling is more strictly turned off if $\Phi_{\mathrm{trans\_CQ}}$ is applied before Φ_{trans_i} compared with applying $\Phi_{\mathrm{trans\_CQ}}$ simultaneously with Φ_{trans_i}. This suggests

that the magnitude of the energy between the three components affects the occurrence of tunneling. In other words, a large energy separation between qubits and the CQ prevents quantum tunneling and turns off the coupling during sweeping of $\Phi_{\mathrm{trans}i}$.

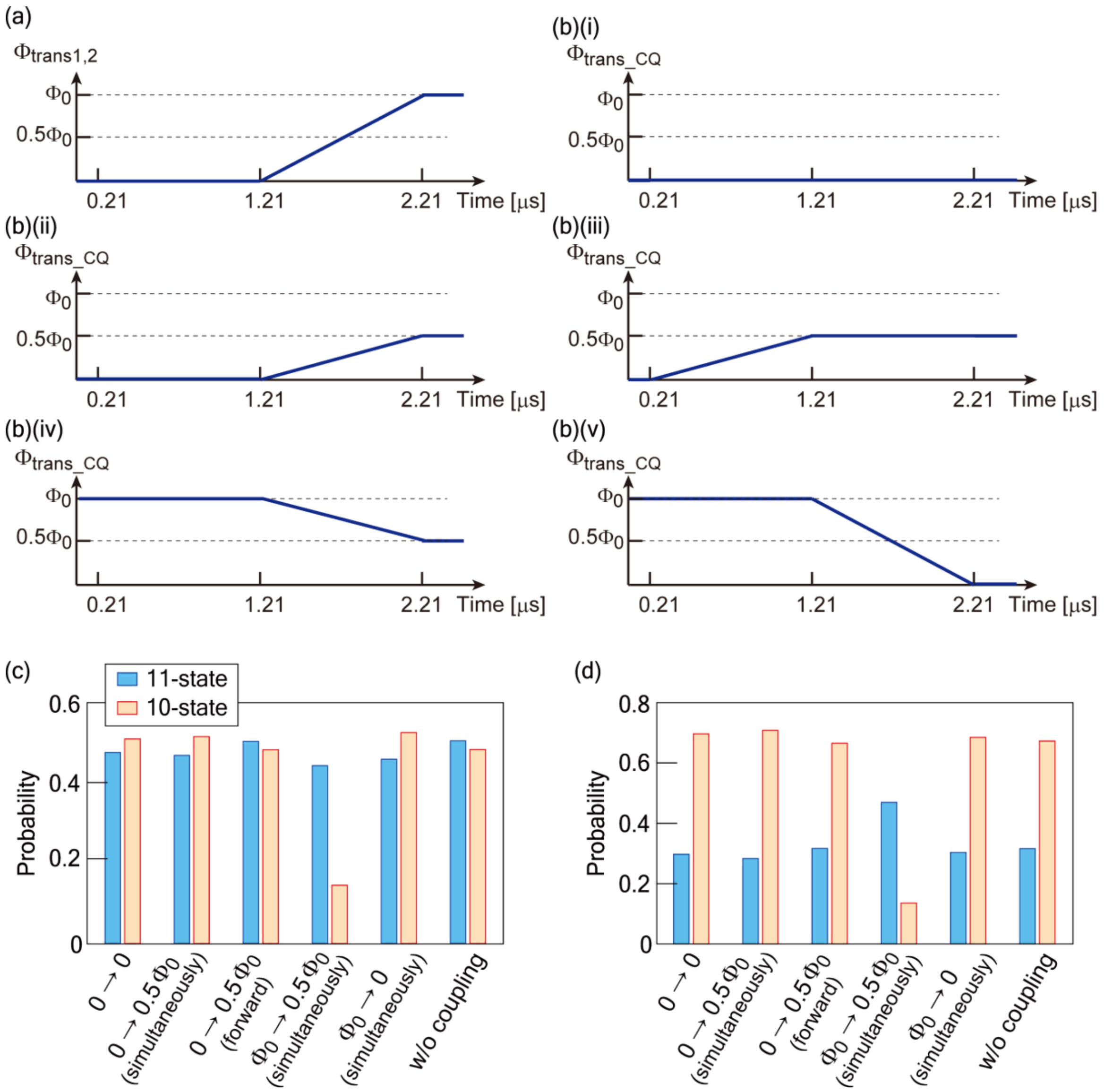

Supplementary Fig. 8 a Time-dependent transverse magnetic flux $\Phi_{\mathrm{trans}1,2}$ applied to qubits Q1 and Q2. **b** Time-dependent transverse magnetic flux $\Phi_{\mathrm{trans\_CQ}}$ applied to the CQ with $\Phi_{\mathrm{trans\_CQ}}$ **(i)** constant at 0, **(ii)** modulated from 0 to 0.5Φ_0 during 1.21–2.21 μs, **(iii)** modulated from 0 to 0.5Φ_0 during 0.21–1.21 μs, **(iv)** modulated from Φ_0 to 0.5Φ_0 during 1.21–2.21 μs, and **(v)** modulated from Φ_0 to 0 during 1.21–2.21 μs. Probability of generating each qubit state in the two qubits coupled by CQ-A after annealing under current conditions of **c** I_{h2} = 0 μA and **d** I_{h2} = -0.2 μA in the JSIM analysis.

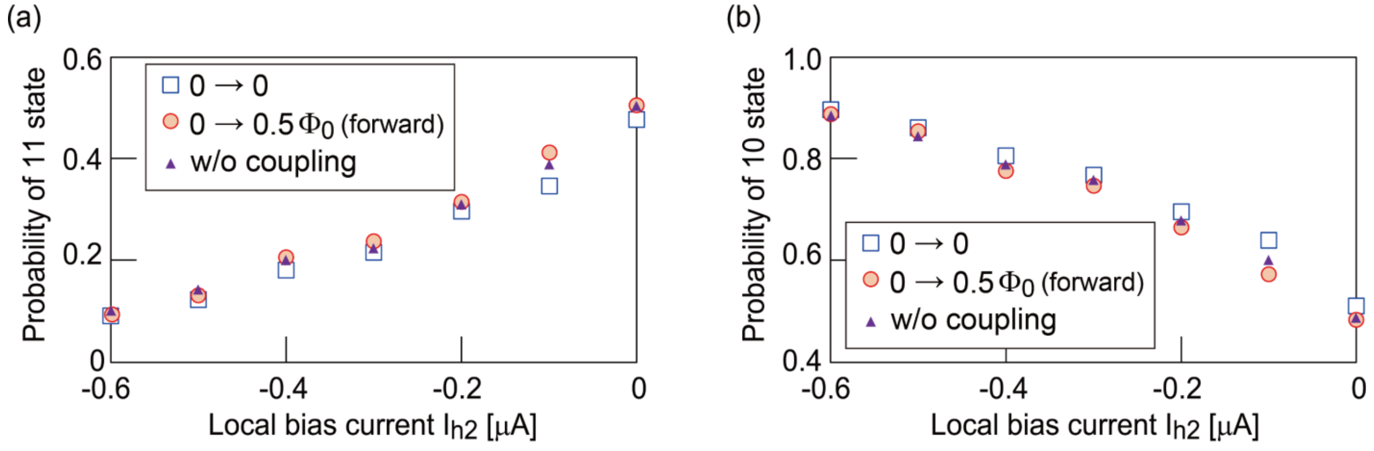

Supplementary Fig. 9 a Probability of the 11 state with respect to modulation of I_{h2}. **b** Probability of the 10 state with respect to modulation of I_{h2}. Φ_{trans_CQ} is kept at 0 or annealing is performed with $0.5\Phi_0$ before the annealing for Q1 and Q2. The w/o coupling conditions indicate that qubits Q1 and Q2 are independently annealed without the interconnection.

Supplementary Note 4: Reliable connection with high yield in flip-chip bonding technology

We establish a flip-chip bonding process using In-Sn reflow soldering, in which 10-µm-high solder bumps are formed uniformly over the entire wafer surface with high reproducibility. Alignment compression using a semi-auto bonder creates reliable connections with high yield. We use In-Sn alloy as the solder because it has a relatively high critical temperature (T_c) of 5 K, which allows us to confirm the superconducting connection between bonded chips by using a helium dewar vessel or conventional refrigerators.

Supplementary Fig. 10 shows the procedure for forming In-Sn solder bumps on the qubit or readout circuits. First, an adhesive region of Au/Ti is formed on the circuit through the lift-off process. Solder bumps are fabricated by dipping the substrate in molten In-Sn alloy in a solder bath. Transition temperature as a function of composition has been reported in the In-Sn binary system[6]. Amorphous alloy bumps (quenching from liquid state) show higher transition temperatures than crystalline alloy bumps. Equilibrium T_c values varied from 3.4 K (near pure In) to 6.6 K (beta phase, 33.5 at.% Sn).

The top and bottom chips are 3.5 × 3.5 and 7.1 × 7.1 mm squares, respectively. The adhesive Au/Ti patterned regions have designed diameters of 40 and 100 µm in the fine pattern area in the qubit (readout) and in the surrounding chip area, respectively. This increases stability in the bump connections and it ensures thermal conductivity between the two chips. Thermal conductivity is crucial because the upper chip is cooled through the bumps when the sample is placed in the refrigerator with the bottom chip in contact with a cold plate (heat sink). Supplementary Figs. 11**a** and 11**b** show optical micrographs of the top chip (qubit) and bottom chip (readout). The black circles correspond to bumps. Bumps are selectively formed on the adhesive region even though the entire chip is immersed in solder solution.

After the bump formation shown in Supplementary Fig. 10**g**, the upper chip is flipped so that its surface with the circuit pattern is downward. The bump positions of top and bottom chips are aligned and the chips are compressed using a flip-chip bonder (BFC-1000, Sony EMCS Corporation). Bonding is performed below 150 °C with a typical bonding force of 10–20 N in the bump area (~10 g/bump), which results in a compression of roughly 10 µm of the total height of the two solder bump depositions. Infrared microscopic inspection of the top and bottom chips indicates that alignment deviation in the plane is less than ±5 µm. As a proof-of-concept demonstration, the bump diameter is 40 µm in the fine pattern in this study, although the bump diameter can be reduced to 20 µm. The rework process is easy because bumps can be made reproducibly by dipping the chip back into the solder solution. The processes after Supplementary Fig. 10**e** can be performed by sputtering and lift-off

method. In this case, the controllability of bump shape will be improved. In addition, due to suppression of heat process, changes in the characteristics of the JJ are avoided. This will be addressed as future work.

In our method, the bottom chip (readout) can be used in common among different flip-chip bonding configurations by changing the circuits on the top chip (qubit), enabling circuits with different functions to be realized. For example, the top chips shown in Supplementary Fig. 11**a** and 11**c** can each be combined with the same bottom chip (Supplementary Fig. 11**b**).

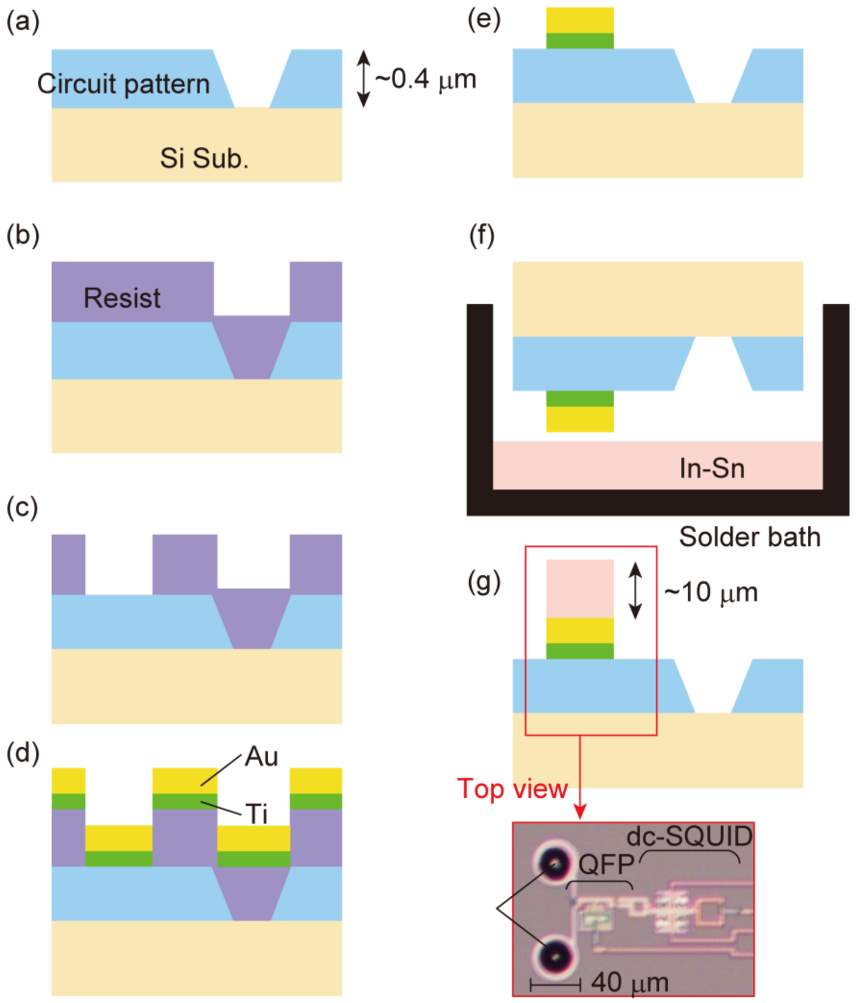

Supplementary Fig. 10 Procedure for forming an In-Sn solder bump. **a** Initial sample structure. For simplicity, the cross section of a circuit consisting of multiple layers is shown for a single pattern. **b** Resist coating, **c** resist patterning by lithography, **d** spattering of Ti/Au layers, **e** lift-off, **f** reflow soldering, and **g** formation of In-Sn bumps.

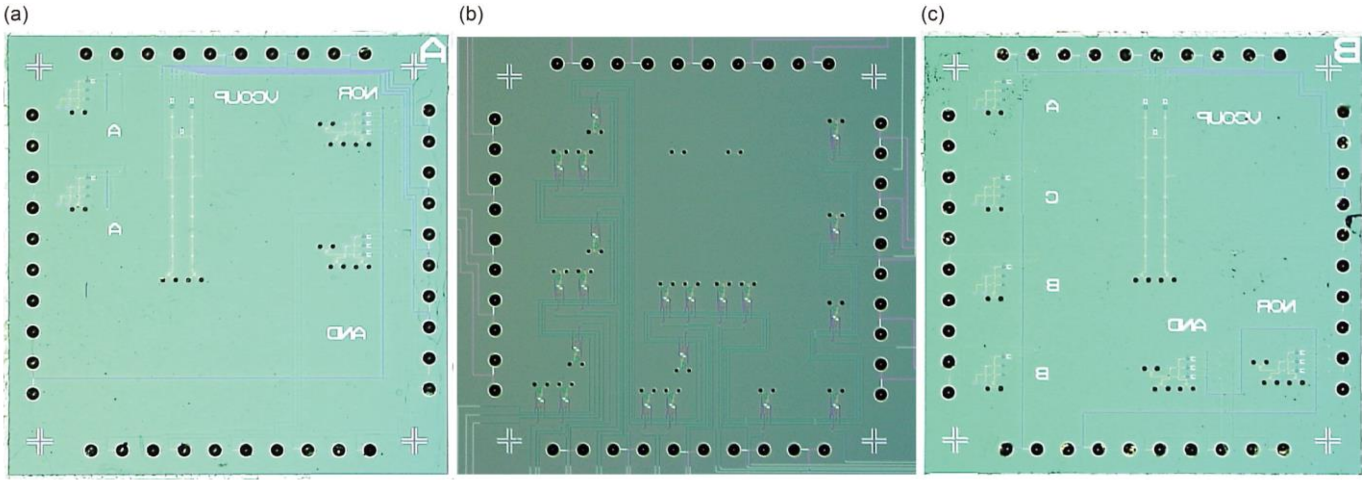

Supplementary Fig. 11 Top-view optical photographs of **a** a 3.5 × 3.5 mm square qubit chip and **b** a 7.1 × 7.1 mm square readout chip. In-Sn solder bumps are formed in the areas visible as black circles. **c** Top-view optical photograph of the 3.5 × 3.5 mm square qubit chip implementing different function compared with that in **a**. The black stuff between the fourth and fifth bumps on the far right is dust.

Supplementary Note 5: Interconnect of gate-type qubits by the CQ

We assume that two gate-type qubits[7,8] couple through the CQ, as shown in Fig. 8**a**. The figure reveals that transmon qubits interact through internal coupling comprising the superconducting path (for example, TiN and Nb). Resonators for readout are not illustrated in the figure to focus on the qubit coupling. Specifically, we consider the transmon qubits formed around the edge of different chips (Qubit Chip-1 and Qubit chip-2). Qubit chip-1, Qubit chip-2, and CQ chip are aligned so that the pattern of each transmon qubit and the main loop in the CQ overlap partially with each other (Fig. 8**b**). The ground planes of Qubit chip-1, Qubit chip-2, and CQ chip are connected through bumps by flip-chip bonding. Figure 8**c** shows the equivalent circuit. Sunada et al.[9] analyzed external coupling between a transmon qubit and the environment. In that study, a Josephson junction (JJ) was replaced by an AC current source for analyzing the real part of the admittance in the finite element model. We focus on verifying the possible interaction between transmon qubits coupled by the CQ. In the simulation, a Josephson integrated circuit simulator (JSIM)[10] is used. Through evaluating the internal current ($I_{\mathrm{p\_Q1}}$) in Q1, we examine whether a change of status in Q2 affects Q1 via the CQ. The possibility of interaction between Q1 and Q2 via the CQ is simulated using the equivalent circuit model shown in Fig. 8**c**. Here, the energy of the gate-type qubit is in the zero-point oscillation state. We consider the interconnection under conditions that do not disturb this state. For the proof-of-concept demonstration, we assume that Q1 and Q2 are superconducting flux Xmon qubits[11]. Each qubit and the CQ have M of 2.2 pH. We consider an internal current flow of 10 nA (net value is 0 over time) in Q2 that is smaller than the zero-point oscillation (15 nA) with a resonance frequency of 5.8 GHz. To simulate this, Q2 is replaced by an AC current source[9]. In this case, the state change induced in Q1 is analyzed by evaluating the internal current ($I_{\mathrm{p\_Q1}}$). The resonance frequency component of $I_{\mathrm{p\_Q1}}$ is modulated with $\Phi_{\mathrm{trans\_CQ}}$ induced by $I_{\mathrm{trans\_CQ}}$ (Supplementary Fig. 12**a**). In particular, $I_{\mathrm{p\_Q1}}$ is suppressed with $\Phi_{\mathrm{trans\_CQ}}$ of $0.5\Phi_0$, which deforms the energy potential of the CQ to a single-well shape. Supplementary Figure 12**b** shows spectra of $I_{\mathrm{p\_Q1}}$. The amplitude at the resonant frequency for $I_{\mathrm{p\_Q1}}$ is modulated by $\Phi_{\mathrm{trans\_CQ}}$, indicating that it may be possible to switch coupling status between Q1 and Q2. The magnitude of the coupling is comparable to the coaxial cable interconnection with the same L in the CQ. Thus, the CQ acts as the interconnection with a switching functionality in the qubit coupling. The on/off ratio in the switching depends on L_{CQ} (Supplementary Fig. 12**c**).

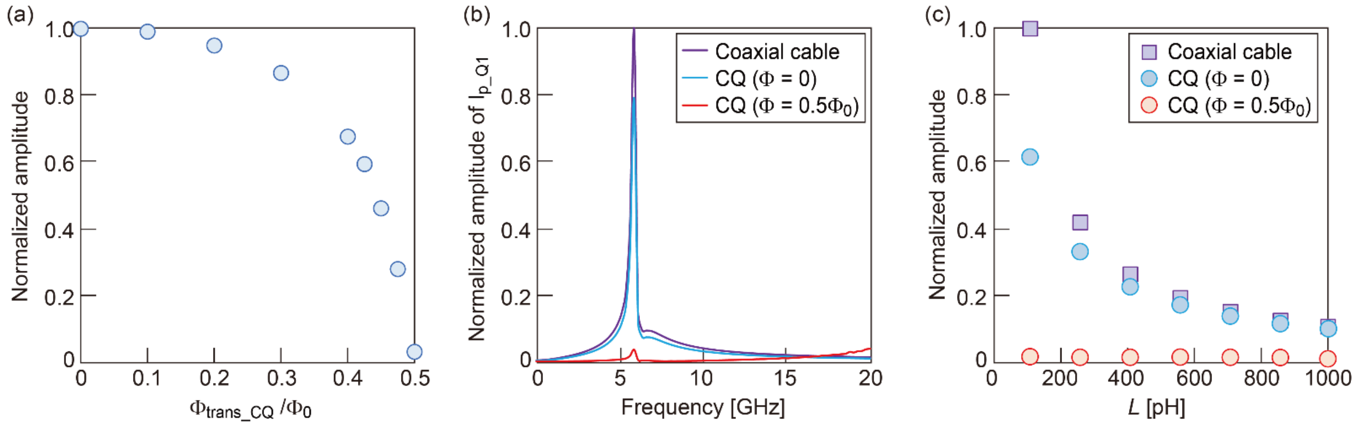

Supplementary Fig. 12 a Analysis of the interconnection in the Xmon qubit. In this model, the JJ is replaced by the SQUID in Fig. 8**c**. Q2 is replaced by an AC current source at 10 nA with a frequency of 5.8 GHz. **a** I_{p_Q1} modulation with respect to Φ_{trans_CQ}. **b** Frequency spectrum of I_{p_Q1} for the CQ with L of 258 pH. **c** Switching characteristics of I_{p_Q1} with respect to L_{CQ}. For reference, we also calculate the model where the Xmon qubit is connected via a coaxial cable instead of the CQ.

References

1. Saida, D. et al. Characterization of energy potential in tunable rf-SQUIDs with the classical regime toward precise design of superconducting flux qubit. *Jpn. J. Appl. Phys*. **60**, 060906 (2021).
2. Saida, D. *et al.* Experimental demonstrations of native implementation of Boolean logic Hamiltonian in a superconducting quantum annealer. *IEEE Trans. Quant. Eng*. **2**, 3103508-3103515 (2021).
3. Whitfield, J. *et al.* Ground-state spin logic, *EPL*, **99**, 57004 (2012).
4. Harris, R. *et al.* Experimental investigation of an eight-qubit unit cell in a superconducting optimization processor. *Phys. Rev. B* **82**, 024511 (2010).
5. Fourie, C. *et al.* Three-dimensional multi-terminal superconductive integrated circuit inductance extraction. *Supercond. Sci. Tech*. **24**, 125015 (2011).
6. Merriam, M. and Herzen, M. Superconductivity in the Indium-Tin System. *Phys. Rev.* **131**, 637 (1963).
7. Hertzberg, J. *et al*. Laser-annealing Josephson junctions for yielding scaled-up superconducting quantum processors. *NPJ Quantum Inf.* **7**, 129 (2021).
8. Sheldon, S. *et al*. Procedure for systematically tuning up cross-talk in the cross-resonance gate. *Phys. Rev. A*. **93**, 060302 (2016).
9. Sunada, Y. *et al*. Fast Readout and Reset of a Superconducting Qubit Coupled to a Resonator with an Intrinsic Purcell Filter. *Phys. Rev. Appl*. **17**, 044016 (2022).

10. Fang, E. S. & Van Duzer, T. A Josephson integrated circuit simulator (JSIM) for superconductive electronics application. in *Proc. Ext. Abstr. 2nd Int. Supercond. Electron. Conf.* 407 (1989).

11. Barends, R. et al. Coherent Josephson Qubit Suitable for Scalable Quantum Integrated Circuits. *Phys. Rev. Lett.* **111**, 080502 (2013).
